# Studies on antidiarrheal and laxative activities of aqueous-ethanol extract of *Asphodelus tenuifolius* and underlying mechanisms

**DOI:** 10.1186/s12906-019-2740-0

**Published:** 2019-11-11

**Authors:** Naveed Aslam, Khalid Hussain Janbaz

**Affiliations:** Department of Pharmacology, Faculty of Pharmacy, Bahauddin Zakariya University, Multan, 60800 Pakistan

**Keywords:** Asphodelaceae, Ca^+ 2^ channel, Spasmolytic, Spasmogenic

## Abstract

**Background:**

*Asphodelus tenuifolius* Cav. (Asphodelaceae) has traditional reputability in treatment of diarrhea and constipation but no scientific study has been reported for its gastrointestinal effects. Present study was conducted to evaluate antidiarrheal and laxative activities of the plant.

**Methods:**

Aqueous-ethanol crude extract of *Asphodelus tenuifolius* (At.Cr) was subjected to phytochemical screening and liquid-liquid fractionation. In vivo studies of charcoal meal intestinal transit test, antidiarrheal activity against castor oil induced diarrhea and laxative activity were performed in mice. In vitro experiments were conducted upon rabbit jejunum preparations using standard tissue bath techniques.

**Results:**

Phytochemical screening indicated presence of alkaloids, anthraquinones, flavonoids, saponins, steroids, tannins and phenols in At.Cr. In charcoal meal intestinal transit test, At.Cr increased (*p* < 0.001) intestinal motility at 100 mg/kg dose, but decreased (*p* < 0.001) it at 500 mg/kg dose, when compared to the control group. At.Cr (300–700 mg/kg) provided protection from castor oil induced diarrhea in mice, which was significant (*p* < 0.001) at 500 and 700 mg/kg doses, as compared to the saline treated control group. At.Cr (50 and 100 mg/kg) enhanced total and wet feces counts in normal mice, as compared to saline treated control. In jejunum preparations, At.Cr inhibited spontaneous, K^+^ (80 mM) and K^+^ (25 mM) mediated contractions, similar to verapamil. Pre-incubation of jejunum preparations with At.Cr resulted in rightward nonparallel shift in Ca^+ 2^ concentration response curves, similar to verapamil. The spasmolytic activity was concentrated in ethylacetate fraction. Aqueous fraction exhibited spasmogenicity upon spontaneous contractions, which was blocked in presence of verapamil, but remained unaffected by other tested antagonists.

**Conclusion:**

The *Asphodelus tenuifolius* crude extract possesses gut modulatory activity, which may normalize gut functions in diarrhea and constipation. The spasmolytic activity of the extract was found to be mediated through Ca^+ 2^ channel blocking action. The spasmogenic activity, found partitioned in aqueous fraction, possibly involves Ca^+ 2^ influx through voltage gated Ca^+ 2^ channels. The study supports ethnic uses of the plant in diarrhea and constipation.

## Background

*Asphodelus tenuifolius* (Cav.) belongs to family Asphodelaceae. The vernacular names of the plant include onion weed (English), Piazi (Urdu), Barwang, Tazai (Arabic) and Dangro, Bokhat, Piazi (Hindi). It is a common weed of Mediterranean and Asian countries [1, 2].

*Asphodelus tenuifolius* is used in cooking as vegetable or added to other dishes for enhancing taste; seeds are used in bread making and sprinkled over dates as elegance [2]. The plant is also used in traditional medicine as antidiarrheal, antipyretic, antidiabetic, aphrodisiac, antitussive [3, 4], antihypertensive [5, 6], hepatoprotective [7], and for treatment of edema, inflammations, constipation, piles and skin problems [1, 8].

Phytochemicals belonging to classes of anthraquinones, alkaloids, flavonoids, phenols, steroids and tannins are reported in the plant extracts [9]. The separated and identified phytochemicals from the plant include apigenin, asphorins, caffeic acid, chrysoeriol, luteolin, luteolin glucoside, rutin, feruloyltyramine, vanillin, β-sitosterol, β-sitosterol glucosides, stigmasterol, dimethoxynaphthalene, chrysophanol, fallacinal, hexadecanoic acid, 3-hydroxybenzoic acids, 1-triacontanol, 1-octacosanol, triacontanoic acid and tetracosanoic acid [9–13]. The seeds were found to contain triglycerides and fucosterol [14].

Scientific studies on the plant extracts and fractions exhibited antioxidant [15], lipoxygenase inhibitory [16], antibacterial, antifungal [9, 17], cardioprotective [18], hypotensive and diuretic [19] activities in various in vivo and in vitro models.

Although *Asphodelus tenuifolius* is traditionally used for the treatment of diarrhea and constipation [1, 8], no pharmacological study has been reported for its gastrointestinal effects. To evaluate potential antidiarrheal and laxative activities of drugs, in vivo studies on mice and in vitro studies upon rabbit isolated jejunum preparations are commonly employed [20–24]. In mice models, charcoal meal test allows study of effects of test material upon gut motility; castor oil model is used to study antidiarrheal potential of test material; while, laxative drugs tend to increase formation of wet diarrheal feces in normal mice. Rabbit isolated jejunum preparations allow study of actions and mechanisms of actions of drugs upon gut smooth muscles. Hence, the present study was aimed to evaluate antidiarrheal and laxative activities of *Asphodelus tenuifolius* extract and fractions using the above mentioned in vivo and in vitro pharmacological assays.

## Methods

### Animals

*Mus musculus* (BALB/c mice, ♂/♀, 20–25 g, 6–8 weeks old, *n* = 79) and *Oryctolagus cuniculus* (rabbit, local breed, ♂/♀, 1.5–2.0 kg, 4–6 months old, *n* = 10) were used. The animals were maintained in cages at 25 ± 2 °C temperature and 12 h light on-off cycles. The animals were having continuous access to standard pellet feed (Shameem Animal Feed, Bahawalpur, Pakistan) and drinking water. The animals were bred and maintained at the animal house of the Faculty of Pharmacy, Bahauddin Zakariya University, Multan, Pakistan. Feed, but not water, was withdrawn 12 h prior to commencement of experiments. Method of cervical dislocation was used to euthanize the animals.

### Chemicals

Analytical grade chemicals were used in experiments. Acetylcholine chloride, atropine sulphate, potassium chloride (KCl), magnesium chloride (MgCl_2_), verapamil hydrochloride, pyrilamine maleate, ethylenediaminetetracetic acid (EDTA), carboxymethylcellulose sodium, castor oil, gallic acid, quercetin and loperamide hydrochloride were of Sigma Chemicals (St. Louis, USA) origin. Sodium chloride (NaCl), sulphuric acid (H_2_SO_4_), hydrochloric acid (HCl), charcoal and sodium nitrite (NaNO_2_) were procured from BDH Laboratory Supplies, Poole, UK. Glucose, ethanol, methanol, calcium chloride (CaCl_2_), magnesium sulphate (MgSO_4_), monopotassium phosphate (KH_2_PO_4_), monosodium phosphate (NaH_2_PO_4_), sodium carbonate (Na_2_CO_3_), sodium bicarbonate (NaHCO_3_) and dimethyl sulfoxide (DMSO) were procured from Merck, Darmstadt, Germany. VWR International Ltd. Poole, UK was supplier of ethylacetate.

### Plant material collection, extraction and fractionation

Whole flowering and fruiting plants of *Asphodelus tenuifolius* were collected from Cholistan desert (29.34798N 72.15702E), Punjab, Pakistan. As the plant is not amongst endangered species, therefore, no written consent was required for its collection. The plant material was verified by expert taxonomist (Dr. Zafar Ullah Zafar, Institute of Pure and Applied Biology, Bahauddin Zakariya University, Multan, Pakistan) and a sample was submitted in herbarium of the institute against voucher number Fl-PK-50-3. The plant material was subjected to shade drying at room temperature for 2 weeks, grounded to rough powder using electric grinder and extracted with aqueous-ethanol (30:70 v/v) by process of maceration at ambient temperature for 72 h and filtered. Maceration of the marc with fresh solvent was repeated twice more; all the three filtrates were combined and subjected to evaporation in rotary evaporator (Rotavapor, Buchi, Switzerland) under reduced pressure (− 730 mmHg) at 37 °C to obtain *Asphodelus tenuifolius* crude extract (At.Cr) [19]. Percentage yield of At.Cr was 9.20% w/w.

The crude extract (40 g) was suspended in distilled water (80 ml), extracted in succession with three aliquots of petroleum ether (3 × 80 ml) and then ethylacetate (3 × 80 ml) in separating funnel for solvent-solvent fractionation. The respective petroleum ether and ethylacetate portions were evaporated in rotary evaporator to obtain petroleum ether fraction (At.Pe) and ethylacetate fraction (At.Ea). The remaining aqueous layer was subjected to freeze drying to get aqueous fraction (At.Aq). The respective yields for At.Pe, At.Ea and At.Aq were calculated 3.52, 5.22 and 85.70% w/w of the crude extract. At.Pe was not studied due to limited availability and poor solubility. The crude extract and fractions were stored at − 4 °C until used.

### Administration of extract and fractions

The extract was suspended in normal saline and administered to mice through oral gavaging [25] in 10 ml/kg volumes. For in vitro experiments, At.Aq was dissolved in distilled water; At.Cr and At.Ea were dissolved in 10% DMSO to make 300 mg/ml stock solution, which were further diluted with distilled water and added to organ bath in cumulative manner to obtain final bath concentrations (0.01–10.0 mg/ml). The selection of doses and concentrations used in experiments was based upon previous studies as well as screening in our laboratory. The solvents as used were without any significant effect upon isolated tissue preparations.

### Phytochemical studies

The At.Cr was screened phytochemically for possible presence of alkaloids, flavonoids, saponins, tannins, phenols, steroids and anthraquinones by using already reported methods [26, 27]. Appearance of yellowish-brown precipitation or coloration following addition of Mayer’s reagent or Drangendroff’s reagent to HCl treated extract solution indicated presence of alkaloids in it. Appearance of froth upon vigorous shaking of the extract aqueous solution in a test tube indicated saponins presence. Appearance of green, purple or blue-black coloration upon treating the extract solution with 1% FeCl_3_ solution showed existence of tannins and phenolic compounds. Appearance of yellow coloration indicated flavonoids existence in the extract upon mixing AlCl_3_ reagent with the extract solution. The extract solution in chloroform was made to contact with concentrated H_2_SO_4_ in a test tube, presence of steroids was indicated upon appearance of red color in the upper chloroform layer and greenish fluorescence in bottom H_2_SO_4_ layer. Appearance of pink or red color upon mixing the extract solution in chloroform with 10% ammonia solution depicted anthraquinones existence.

### Total phenolic and flavonoid contents

Total phenolic and flavonoid contents in the extract were estimated by already reported methods [27]. For phenolic content estimation, 0.5 ml of test solution (1 mg/ml) was mixed with 0.5 ml Folin-Ciocalteu reagent. After 5 min, Na_2_CO_3_ solution (20%; 1 ml) was added to it, mixed and allowed to stand for 10 min in dark. Subsequent to centrifugation, the absorbance of the supernatant was measured at 730 nm.

Flavonoids content of the extract was estimated by AlCl_3_ colorimetric method. Briefly, dilute ethanol solution of the test material (3.7 ml) was mixed and incubated with NaNO_2_ solution (0.5 M; 0.15 ml) and AlCl_3_ solution (0.3 M; 0.15 ml) for 5 min. Then NaOH solution (1 M; 1 ml) was mixed and absorbance at 506 nm was measured.

The phenol and flavonoid contents of the extract were determined from standard concentration absorbance curves for gallic acid and quercetin, respectively.

### Charcoal meal intestinal transit test

Movement of charcoal meal through small intestine of mice was assessed using slight modifications of already reported method [20]. The mice of either sex were randomized to six groups, with five animals in each group. The control group was treated with normal saline (10 ml/kg, p.o.). The extract was given to four treatment groups at respective oral doses of 50, 100, 500 and 700 mg/kg. Two standard groups were subjected to treatments with loperamide (10 mg/kg, p.o.) and carbachol (1 mg/kg, p.o.). Subsequently, 15 min after the above treatments, all the groups received oral doses (0.2 ml/mouse) of charcoal meal (suspension of 5% charcoal in distilled water containing 2% sodium carboxymethylcellulose). After 30 min of charcoal meal administration, the mice were scarified through cervical dislocation [20, 21], small intestines were rapidly removed and distance travelled by the charcoal meal in small intestine as percent of total small intestine length was measured in all animals for comparison with control group.

### Antidiarrheal activity

The mice were randomly divided to five groups, with five animals in each group. First group designated as diarrheal control group was treated orally by saline (10 ml/kg). The second to fourth treatment groups received extract at 300, 500 and 700 mg/kg doses, respectively. The fifth group, designated as standard group, received loperamide (10 mg/kg, p.o.). After 60 min of the treatments, all the groups were given castor oil (10 ml/kg, p.o.). Animals were then individually placed in filter paper lined polycarbonate cages. Subsequently, numbers of diarrheal feces were counted for all groups at 4 h of the last treatment for comparison to saline treated control group [21].

### Laxative activity

The mice of either sex were randomized into four groups, with six animals in each group. The control group was treated with normal saline (10 ml/kg, p.o.). The two treatment groups received 50 and 100 mg/kg oral doses of At.Cr. The fourth standard group was treated with carbachol (1 mg/kg, p.o.). After treatments, the mice were individually placed in filter paper lined polycarbonate cages and monitored for 6 h to count number of total feces and wet feces. The increase in total and wet feces counts compared to the control group showed laxative activity [21].

### In vitro experiments upon jejunum preparations

Rabbits were scarified by cervical dislocation; jejunums were dissected out and cut into about 2 cm long cylindrical pieces while keeping in oxygenated Tyrode’s solution in Petri plates. Tyrode’s solution was having composition: MgCl_2_ = 1.05 mM, NaCl = 136.90 mM, NaHCO_3_ = 11.90 mM, NaH_2_PO_4_ = 0.42 mM, KCl = 2.68 mM, CaCl_2_ = 1.80 mM and glucose = 5.55 mM (pH = 7.4). Each jejunum segment was cleaned off from adhering tissues and hanged in Tyrode’s solution in 15 ml tissue bath. Contractions were recorded using isotonic transducer (MLT0015) attached with Power Lab® data Acquisition System (AD Instruments, Sydney, Australia). The bathing solution was kept at 37 °C by thermo-circulator and gassed by steady stream of carbogen (95% O_2_ with 5% CO_2_) throughout experiment. The preparation was then subjected to preload (1 g) and allowed to establish its spontaneous contractions, while bathing solution was replaced with fresh Tyrode’s solution at 10 min intervals. Subsequently, the preparation was repeatedly exposed to acetylcholine (0.3 μM) and washings till two identical consecutive contractile responses were attained. The test material was then added to tissue bath in cumulative fashion (0.01–10.0 mg/ml) to assess its effects upon spontaneous contractility of jejunum preparations. The test material exhibiting contractile activity upon spontaneous contractions was further investigated in presence of different antagonists. To study mechanism of spasmolytic activity, the test material was added to preparations pre-contracted with either KCl (80 mM) or KCl (25 mM). The relaxant response of test materials was calculated as percent of initial contractility prior to addition of first dose of test material [22].

The Ca^+ 2^ channel blocking activity of test material was authenticated by stabilizing the rabbit jejunum preparations in Tyrode’s solution, followed by exposure to CaCl_2_ free Tyrode’s solution containing EDTA (0.1 mM) for 30 min to remove Ca^+ 2^ from the preparation. Then the above mentioned solution was further replaced with CaCl_2_ free Tyrode’s solution containing high K^+^ contents having composition: glucose = 5.55 mM, KCl = 50 mM, MgCl_2_ = 1.05 mM, NaCl = 91.04 mM, NaHCO_3_ = 11.90 mM, NaH_2_PO_4_ = 0.42 mM and EDTA = 0.1 mM (pH = 7.4). After equilibration, CaCl_2_ solution was added in cumulative manner to construct concentration response curves (CRCs) for Ca^+ 2^. On attaining the reproducible CRCs, the rabbit jejunum preparations were incubated with different concentrations of the test material (0.1 and 0.3 mg/ml) for 60 min and Ca^+ 2^ CRCs were again obtained in presence of test material [22].

### Statistical analysis

The data values were presented as mean ± standard error of mean (SEM) or half maximum effective concentration (EC_50_) with confidence interval (CI) 95%. Software GraphPad Prism (GraphPad, USA) was used for data analysis and construction of graphs. The statistical tool applied for comparison of data groups was ANOVA along with Dunnett’s test with level of significance *p* < 0.05.

## Results

### Phytochemical analysis, phenol and flavonoids contents

Phytochemical analysis indicated presence of alkaloids, anthraquinones, flavonoids, saponins, steroids, tannins and phenols in the extract. The crude extract was found to contain 57.67 ± 4.48 (*n* = 3) mg/g total phenols and 35.00 ± 1.73 (*n* = 3) mg/g flavonoids based upon respective gallic acid and quercetin equivalents.

### Charcoal meal intestinal transit

**Fig. 1:**
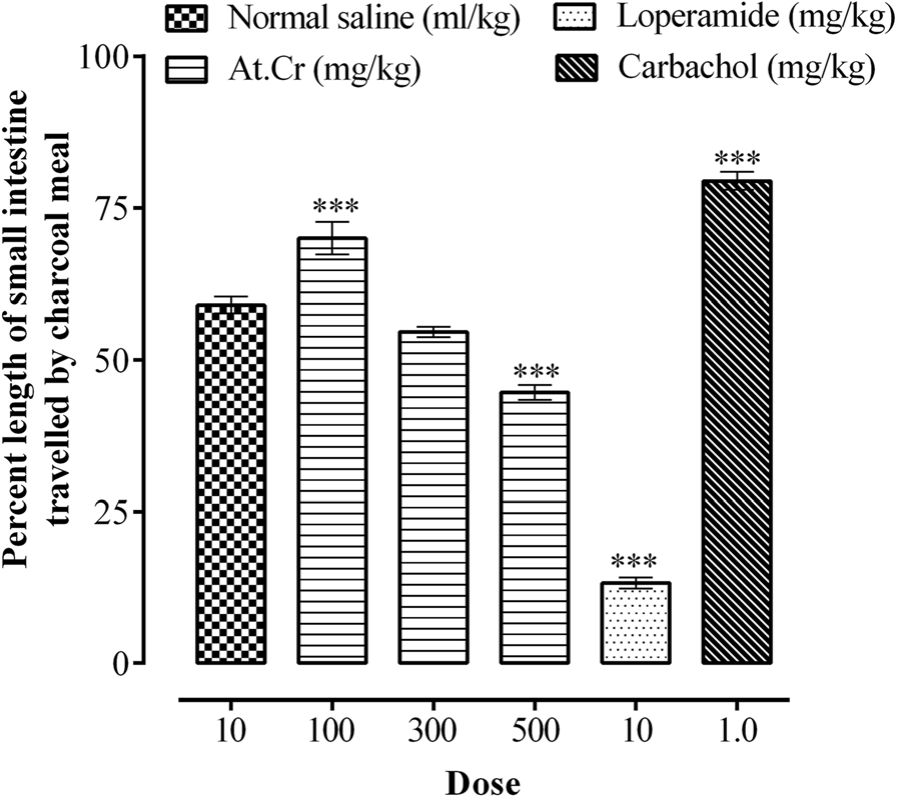
Effects of *Asphodelus tenuifolius* crude extract (At.Cr), loperamide and carbachol upon charcoal meal transit in small intestine of mice. Values are mean ± SEM (*n* = 5). ***p < 0.001 versus saline treated control group

The charcoal meal transit through small intestine of mice as percent of total length of small intestine in various groups is shown in Fig. 1. The charcoal meal small intestinal transit was found to be 59.02 ± 1.36% (*n* = 5) in saline treated control group. Oral administration of At.Cr (100 mg/kg) resulted in significant enhancement (*p* < 0.001 versus control) of intestinal transit of charcoal meal, with resulting value of 70.02 ± 2.69% (*n* = 5) of total small intestinal length. However, administration of At.Cr at 300 mg/kg dose caused insignificant decrease (*p* > 0.05 versus control) in charcoal meal travel through small intestine, which was found to be 54.62 ± 0.87% (*n* = 5) of total small intestine length. Moreover, charcoal meal intestinal transit in group of mice treated with At.Cr (500 mg/kg) was found to be 44.64 ± 1.23% (*n* = 5) of total small intestinal length, which was found to be significantly decreased (*p* < 0.001) as compared to the control group. Furthermore, loperamide treatment resulted in significant decline (*p* < 0.001) and carbachol treatment resulted in significant (*p* < 0.001) enhancement in charcoal meal intestinal transits with respective values of 13.24 ± 0.93% (*n* = 5) and 79.48 ± 2.50% (*n* = 5) of small intestine lengths.

### Antidiarrheal activity

**Fig. 2:**
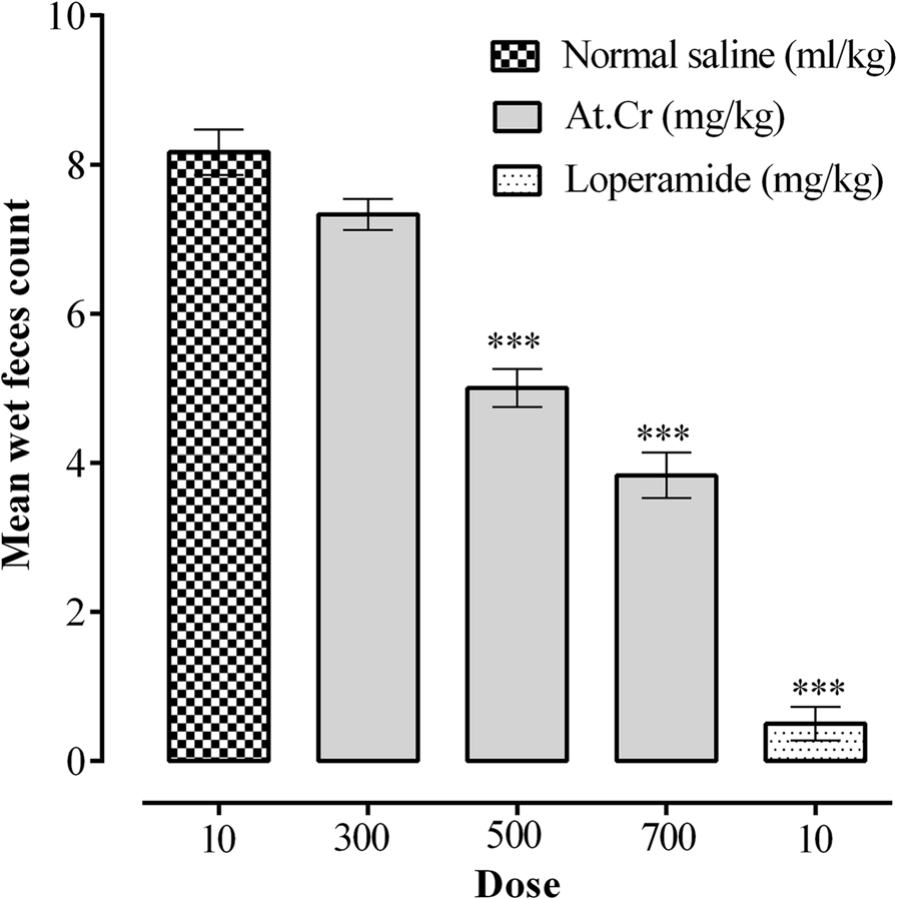
Antidiarrheal effects of *Asphodelus tenuifolius* crude extract (At.Cr) and loperamide against castor oil induced diarrhea in mice. Values are mean ± SEM (*n* = 5). ***p < 0.001versus saline treated control group

Administration of castor oil to saline treated diarrheal control group produced diarrhea in all mice with mean wet feces count of 8.20 ± 0.31 (*n* = 5). Administration of castor oil to mice pretreated with At.Cr (300 mg/kg) resulted in 7.40 ± 0.24 (*n* = 5) wet feces in mice, but the reduction in diarrheal feces was insignificant (*p* > 0.05) as compared to diarrheal control group. However, At.Cr (500 and 700 mg/kg) treatments caused significant reduction (*p* < 0.001) in castor oil induced diarrheal feces with mean wet fecal counts of 5.00 ± 0.32 (*n* = 5) and 3.80 ± 0.37 (*n* = 5), respectively, as compared to diarrheal control group. Loperamide (10 mg/kg) treatment reduced castor oil induced diarrheal feces count to 0.50 ± 0.22 (*n* = 5, *p* < 0.001) in comparison with control group (Fig. 2).

### Laxative activity

**Fig. 3:**
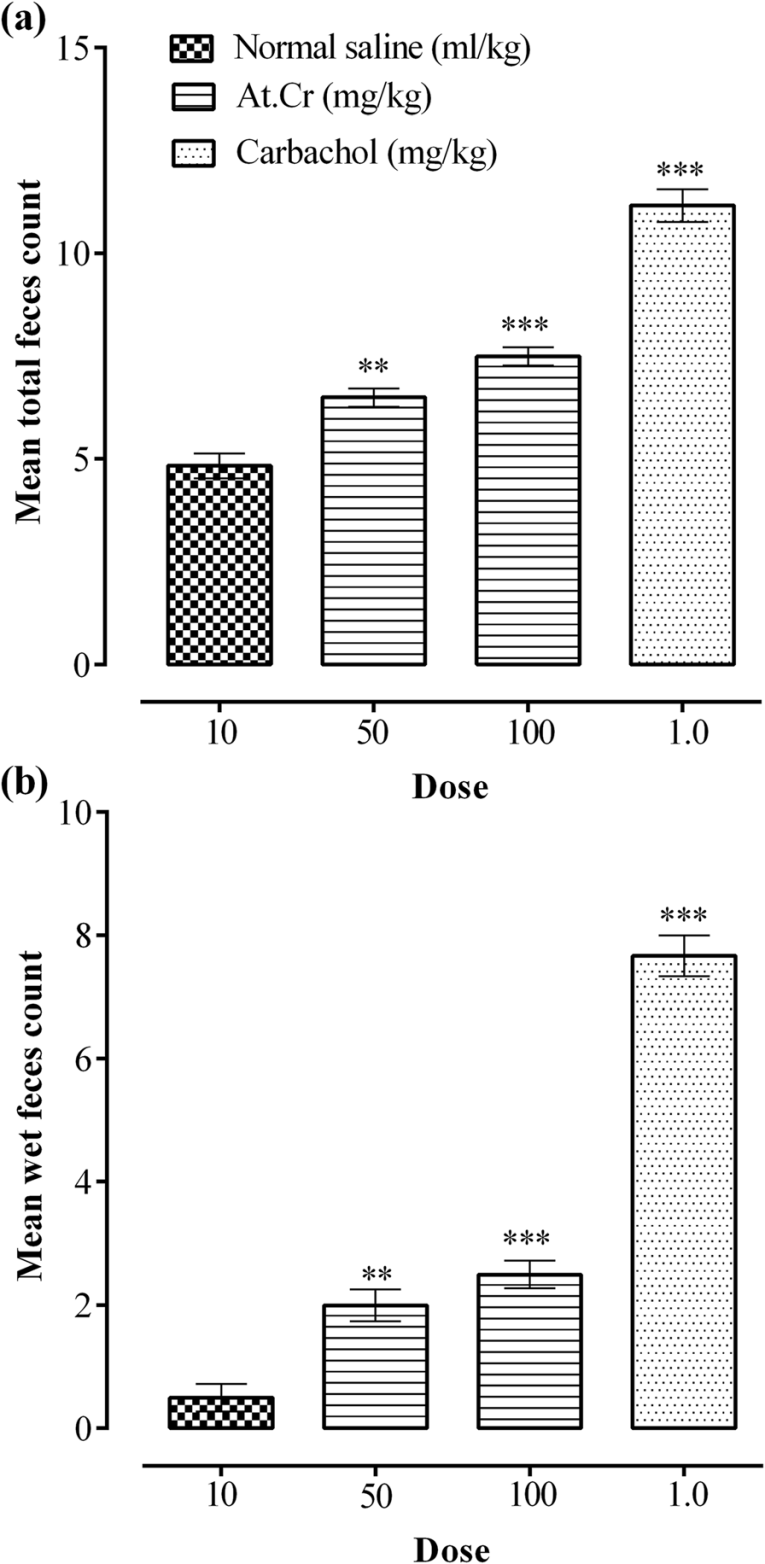
Effects of *Asphodelus tenuifolius* crude extract (At.Cr) and carbachol upon (**a**) total feces and (**b**) wet feces output in mice. **p < 0.01 and ***p < 0.001 versus saline treated control group. Values are mean ± SEM (*n* = 6)

The counts of total and wet feces produced by mice of various groups are plotted in Fig. 3. The extract at 50 mg/kg dose resulted in 6.50 ± 0.22 total feces and 2.00 ± 0.26 wet feces (*n* = 6), as opposed to 4.83 ± 0.31 total feces and 0.50 ± 0.22 wet feces in saline treatment group (*n* = 6), and the increase was significant (*p* < 0.01) in total as well as wet feces counts. Furthermore, the extract at 100 mg/kg dose also caused significant increase (*p* < 0.001) in total and wet feces with respective counts of 7.50 ± 0.22 and 2.50 ± 0.22 (*n* = 6), as compared to saline treated control group. Similarly, carbachol also increased (*p* < 0.001) total feces count to 11.17 ± 0.40 and wet feces count to 7.67 ± 0.33 (*n* = 6).

### In vitro experiments upon jejunum preparations

**Fig. 4:**
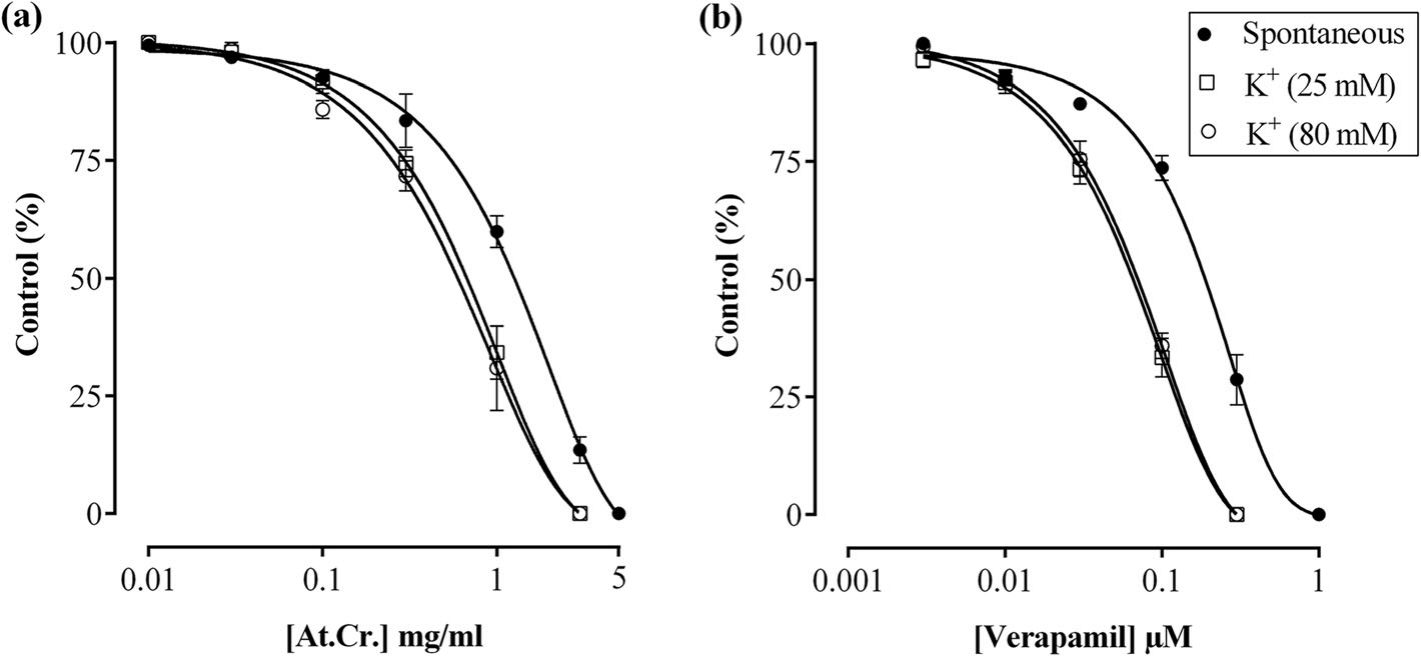
Inhibitory effects of (**a**) *Asphodelus tenuifolius* crude extract (At.Cr) and (**b**) verapamil against spontaneous, K^+^ (25 mM) and K^+^ (80 mM) induced contractions in jejunum preparations. Values are mean ± SEM (*n* = 5)

The spontaneous contractions, K^+^ (80 mM) and K^+^ (25 mM) mediated contractions in rabbits isolated jejunum preparations were relaxed by At.Cr with half maximum effective concentration (EC_50_) values of 1.34 mg/ml (95% CI = 1.11–1.61, *n* = 5), 0.61 mg/ml (95% CI = 0.48–0.76, *n* = 5) and 0.67 mg/ml (95% CI = 0.57–078, *n* = 5), respectively (Fig. 4a). The order of potency of At.Cr in relaxing spontaneous, K^+^ (80 mM) and K^+^ (25 mM) induced contractions was as follows: spontaneous < K^+^ (80 mM) ≈ K^+^ (25 mM). Verapamil, a standard Ca^+ 2^ channel blocking drug also manifested the above stated order of relaxant activities against K^+^ (80 mM), K^+^ (25 mM) induced contractions and spontaneous contractions in jejunum preparations with EC_50_ values of 0.06 μM (95% CI = 0.05–0.07, *n* = 5), 0.06 μM (95% CI = 0.04–0.07, *n* = 5) and 0.17 μM (95% CI = 0.15–0.19, *n* = 5), respectively (Fig. 4b).

**Fig. 5:**
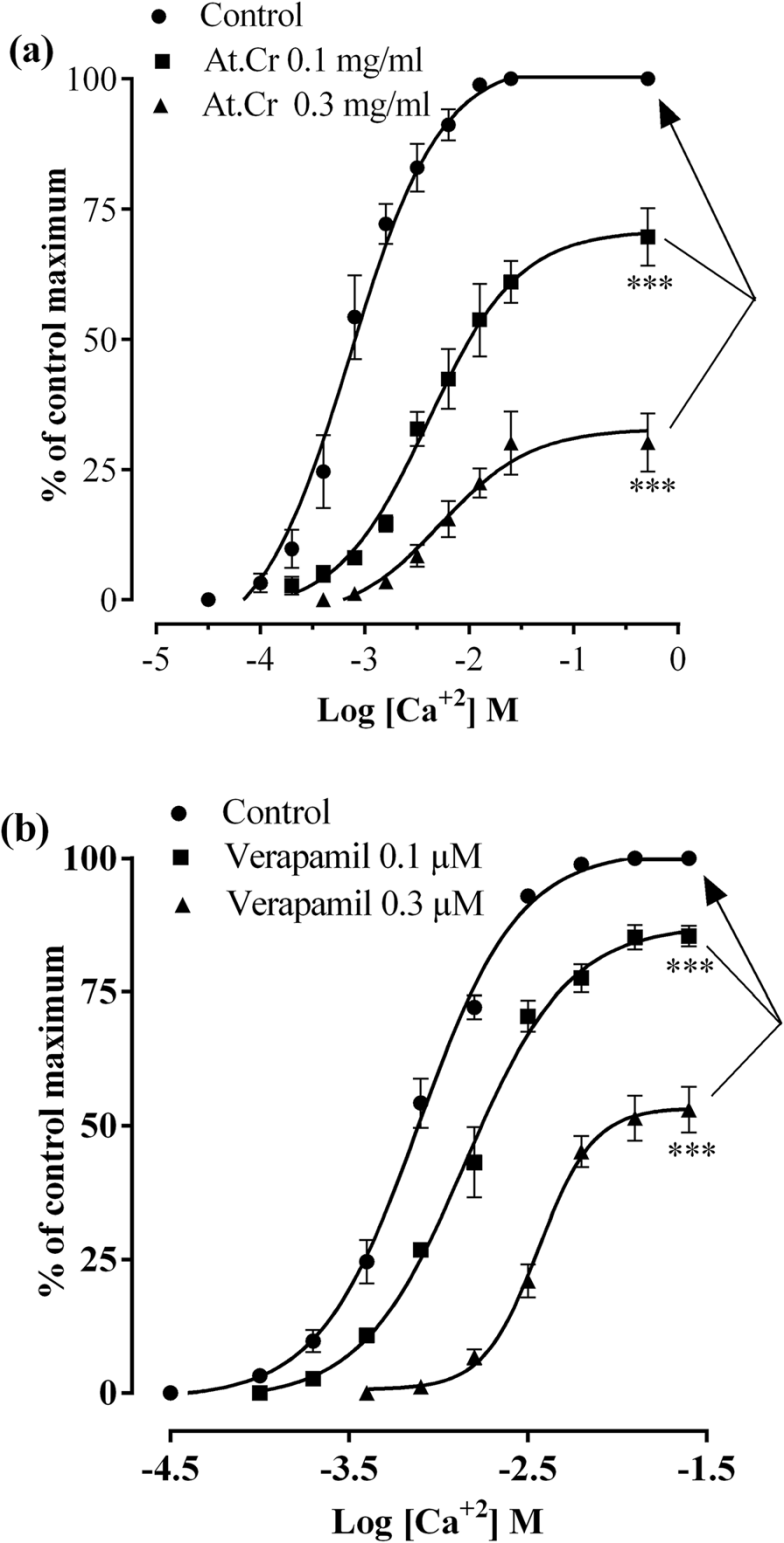
Effects of (**a**) *Asphodelus tenuifolius* crude extract (At.Cr) and (**b**) verapamil upon Ca^+ 2^ concentration response curves in jejunum preparations. ***p < 0.001 as analyzed by two way ANOVA followed by Dunnett’s test. Values are mean ± SEM (*n* = 5)

Furthermore, in jejunum preparations, pretreatment of tissues with At.Cr (0.1 and 0.3 mg/ml) caused shifting of Ca^+ 2^ CRCs rightward in non-parallel fashion while suppressing highest possible response significantly (*p* < 0.001) as compared to the control (Fig. 5a). Similarly, pretreatment with verapamil (0.1 and 0.3 μM) resulted in shifting of CRCs for Ca^+ 2^ in non-parallel manner with decline in highest possible response significantly (*p* < 0.001) in jejunum preparations, as compared to the control response (Fig. 5b).

**Fig. 6:**
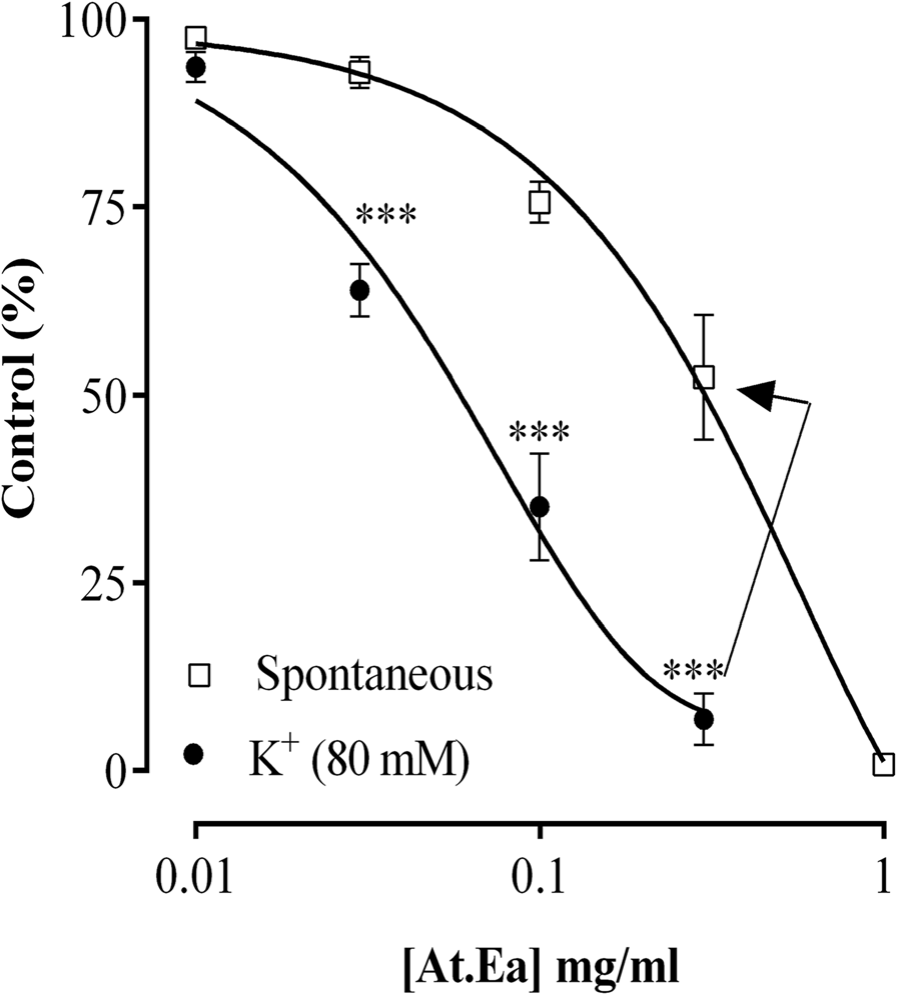
Effects of *Asphodelus tenuifolius* ethylacetate fraction (At.Ea) upon spontaneous contractions and K^+^ (80 mM) induced contractions in rabbit jejunum preparations. ***p < 0.001 as analyzed by two-way ANOVA followed by Dunnett’s test. Values are mean ± SEM (*n* = 5)

**Fig. 7:**
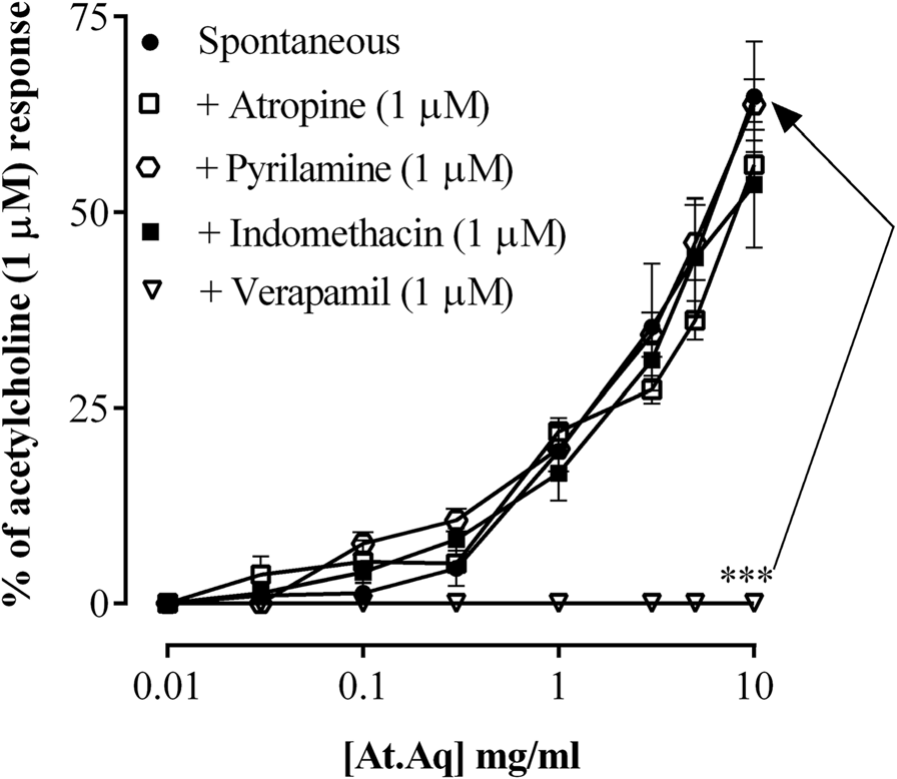
Effects of *Asphodelus tenuifolius* aqueous fraction (At.Aq) upon spontaneous contractions in jejunum preparations alone and in presence of various antagonists. ***p < 0.001 as analyzed by two-way ANOVA with Dunnett’s test. Values are mean ± SEM (*n* = 5)

Furthermore, ethylacetate fraction of the extract (At.Ea) relaxed spontaneous contractions and K^+^ (80 mM) mediated contractions in rabbit isolated jejunum preparations at EC_50_ of 0.26 mg/ml (95% CI = 0.20–0.33, *n* = 5) and 0.05 mg/ml (95% CI = 0.04–0.06, *n* = 5), respectively (Fig. 6). The At.Aq (aqueous fraction of the crude extract) enhanced spontaneous rhythmic contractions in jejunum preparations; which stood uninfluenced in presence of atropine (1 μM), indomethacin (1 μM) or pyrilamine (1 μM), but was completely blocked subsequent to pre-treating the jejunum preparations with 1 μM verapamil (Fig. 7). The At.Aq (0.01–10.0 mg/ml) was without any significant effect upon K^+^ (80 mM) mediated contractions in jejunum preparations.

## Discussion

*Asphodelus tenuifolius* has ethno-botanical repute in management of constipation and diarrhea [3, 4, 8]. Therefore, At.Cr, At.Ea and At.Aq were investigated using in vivo and in vitro animal models to validate the ethnic medicinal repute of the plant in literature.

Oral administration of At.Cr (100 mg/kg) to mice resulted in significant (*p* < 0.001) enhancement of charcoal meal intestinal transit, indicating prokinetic potential of the plant extract. Administration of At.Cr (50 and 100 mg/kg) also caused increase in production of total and wet feces in normal mice, thus demonstrated prokinetic and laxative activities, in manner similar to carbachol (1 mg/kg). However, at higher oral doses, the At.Cr (300 and 500 mg/kg) reduced charcoal meal travel through small intestine of mice, which was found significant (*p* < 0.001) at 500 mg/kg dose, when compared with the saline treated control group. Oral treatments with At.Cr (300, 500 and 700 mg/kg) also manifested prevention from castor oil induced diarrhea in mice, which was found significant (*p* < 0.001) at 500 and 700 mg/kg doses. The castor oil is familiar to induce diarrhea due to its hydrolyzed active product ricinoleic acid, which through increased prostaglandin synthesis and release, alters intestinal water and electrolytes transport and also increase intestinal motility to produce diarrhea [28]. The standard drug loperamide also showed significant (*p* < 0.001) inhibition of charcoal meal intestinal travel and castor oil induced diarrhea in mice. It is known that antidiarrheal effect of loperamide primarily involves decrease in motor activity of intestine [29, 30]. Diarrhea may result from numerous pathological conditions of gastrointestinal tract leading to impaired transport of water and electrolytes or rapid gut motility [31]. The antidiarrheal activity of At.Cr may be attributed to its inhibitory action on intestinal motility. It is also known that the *Asphodelus tenuifolius* extracts possess antioxidant and antibacterial activities [9, 15, 17, 18], which may also provide additional contribution in its antidiarrheal potential depending upon etiology of the diarrheal condition.

The rabbit isolated jejunum preparations are viewed appropriate for studying spasmolytic and spasmogenic activities of herbal extracts [23, 24]. The At.Cr, At.Ea and verapamil, when applied in increasing cumulative concentrations to jejunum preparations caused inhibition of spontaneous contractions. The spontaneous contractions of jejunum preparations are mediated upon recurrent depolarization of smooth muscle cell membrane; leading to opening of voltage gated Ca^+ 2^ channels, increased cytosolic Ca^+ 2^ concentrations and activation of contractile machinery of cells [32]. It is already reported that spontaneous contractions of rabbit isolated jejunum preparations are inhibited by plant extracts having Ca^+ 2^ channel blocking or K^+^ channel opening activities [22]. Hence, spasmolytic activity of the extract was further explored for the above stated possible mechanisms by testing it against K^+^ (80 mM) and K^+^ (25 mM) mediated contractions in jejunum preparations. The smooth muscles undergo contractions upon exposure to K^+^ (20–80 mM), chiefly due to activation of voltage dependent Ca^+ 2^ channels [33]. The substances capable of causing relaxation against K^+^ (80 mM) and K^+^ (25 mM) induced contractions in smooth muscles, in equipotent manner, are viewed as voltage gated Ca^+ 2^ channel blockers [22, 34]. In contrast, agents that relax K^+^ (25 mM) mediated contractions in preference over K^+^ (80 mM) mediated contractions are regarded as K^+^ channels openers [22]. As, At.Cr was found almost equipotent in relaxing K^+^ (80 mM) and K^+^ (25 mM) mediated contractions in jejunum preparations, therefore, the relaxant potential of At.Cr may be accounted to its blocking activity at voltage gated Ca^+ 2^ channels, while excluding K^+^ channels opening activity. Similarly, typical Ca^+ 2^ channel blocker (verapamil) also exhibited equal potency in relaxing K^+^ (80 mM) and K^+^ (25 mM) mediated contractions, while being less potent in relaxing spontaneous contractions, in manner identical to At.Cr. The Ca^+ 2^ channel blocking activity of At.Cr was verified as subsequent to treatment of jejunum preparations with At.Cr (0.1 and 0.3 mg/ml), the CRCs for Ca^+ 2^ was shifted toward right with mitigation of maximum response, in manner comparable to verapamil [27].

The ethylacetate fraction of the extract showed potent relaxing activity against K^+^ (80 mM) than spontaneous contractions in jejunum preparations in manner similar to verapamil, which indicated partitioning of Ca^+ 2^ channel blocking constituents into organic fraction. Whereas, aqueous fraction was found to possess contractile activity upon spontaneous contractions in jejunum preparations, which remained unaffected upon pre-treating the preparations with atropine (antimuscarinic drug), indomethacin (prostaglandin synthesis inhibitor) or pyrilamine (antihistamine drug) [35], thus excluding stimulating role of At.Aq at muscarinic receptors, prostaglandin synthesis and histamine receptors in spasmogenic activity of the fraction [32, 36]. However pre-incubating the tissues with verapamil (1 μM) blocked contractile activity of At.Aq, indicating involvement of Ca^+ 2^ channels mediated Ca^+ 2^ influx in spasmogenic activity of At.Aq [36].

Opposite stimulating and blocking activities at same receptors have already been documented in plant extracts [24] and chemicals [37]. Furthermore, presence of spasmogenic activity in aqueous fraction indicates water soluble nature of spasmogenic constituents. The crude extract did not exhibit spasmogenic activity in rabbit jejunum preparations, which indicates dominance of spasmolytic constituents in the crude extract. However, fractionation caused separation of potent spasmolytic constituents in organic fraction, while unmasking spasmogenic constituents in aqueous fraction. Chemical antidiarrheal and laxative drugs are commonly accompanied with side effects linked to their pharmacological activities; antidiarrheal drugs (e.g. loperamide and morphine) tend to cause constipation, while certain drugs used in treatment of constipation (e.g. bisacodyl and serotonergic drugs) are associated with diarrhea and abdominal cramps [38]. Alternative herbal medicine having both spasmolytic and spasmogenic activities, like *Asphodelus tenuifolius*, may be helpful in balancing gut motility while counteracting unwanted depressant or stimulant actions upon gut movements.

The results of phytochemical investigations on At.Cr are in agreement with already reported studies [9–13] indicating presence of alkaloids, anthraquinones, flavonoids, saponins, tannins, phenols and steroids in *Asphodelus tenuifolius*. Previous studies showed that antidiarrheal activity of medicinal plants may be due to alkaloids, flavonoids and tannins, as these phytochemicals were found to decrease gut motility and secretions [39]. In contrast, anthraquinones are reported to exhibit laxative activity due to their ability to increase intestinal motility and secretions [40]. Therefore, presence of these phytochemicals in At.Cr may be responsible for antidiarrheal and laxative activities of *Asphodelus tenuifolius*. Nevertheless, further studies elaborating identification and isolation of phytochemicals responsible for the gastrointestinal effects of the plant are recommended.

## Conclusion

The study concludes that *Asphodelus tenuifolius* crude extract possesses gut modulatory activity. The spasmolytic and antidiarrheal activities of the extract may be mediated through Ca^+ 2^ channel blocking action. The spasmogenic and laxative activities possibly involve influx of Ca^+ 2^ through voltage operated Ca^+ 2^ channels. The study provided pharmacological authentications for ethnic medicinal utilization of *Asphodelus tenuifolius* in diarrhea and constipation.

## Data Availability

The data generated /analyzed during the current study is available from the corresponding author on reasonable request.

## Abbreviations

Ach: Acetylcholine
At.Aq: Aqueous fraction of At.Cr
At.Cr: *Asphodelus tenuifolius* crude extract
At.Ea: Ethylacetate fraction of At.Cr
CI: Confidence interval
CRCs: Concentration response curves
EC_50_: Half maximum effective concentration
h: Hour
min: Minutes
p.o.: per os / orally
